# Quercetin-gavaging quashes anaphylaxis sequela in the peanut sensitised wistars

**DOI:** 10.1186/2045-7022-5-S3-P145

**Published:** 2015-03-30

**Authors:** Lotfollah Behroo, Farideh Shishehbor, Mehri Ghafouriyan Broujerdnia, Forough Namjouyan, Seiyed-Mahmoud Latifi

**Affiliations:** 1 AJUMS, Ahvaz, Iran

## Background

Regarding the large figures of patients affected with potentially life-threatening peanut allergy, the possibility of inevitable occurrence of unexpected exposures and, the paucity of promising acceptable immunotherapy approaches, more applicable and at the same time, safe remedial strategies are necessitated.

Quercetin, a nutritional panacea belonging to “Flavonol” sub-group of the flavonoid family, thanks to its antioxidant/free radical-scavenging, anti-inflammatory and other protective properties has been suggested for therapy of humankind allergic diseases and might be helpful for the treatment of food allergies.

## Objective

The main aim of our study was to investigate the anti-aanaphylactic characteristics of quercetin in a wistar-rat model of peanut allergy.

## Material and methods

40 male wistar rats, 4-6 weeks old at study start, were sensitized with crude peanut herape in the presence of cholera toxin adjuvant. The sensitized rats in the treatment group (n= 10), were treated intragastrically with quercetin, at a dose of 50 mg/Kg.BW over a period of four weeks. Subsequently, some well-known anaphylaxis-determinant variables were measured.

## Results

Following calculations, it was revealed that quercetin has sufficiently abrogated peanut-induced anaphylactic reactions. Accordingly, plasma histamine and total serum IgE levels had been lowered significantly, in treatment group (p< 0.000 and p<0.000, respectively). Additionally, all of the intradermal- and intraperitoneal-challenge test-results were negative in the quercetin-treated animals.

## Conclusions

Our results approved that a naturally-occurring flavonoid, Quercetin, has been efficient enough to curb the peanut allergy complications in a wistar model and can be considered as a secure preventative/prophylactic/herapeutic compound in populations suffering from potentially deathful IgE-mediated food allergies.

